# Engineering of Hollow Mesoporous Silica Nanoparticles for Remarkably Enhanced Tumor Active Targeting Efficacy

**DOI:** 10.1038/srep05080

**Published:** 2014-05-30

**Authors:** Feng Chen, Hao Hong, Sixiang Shi, Shreya Goel, Hector F. Valdovinos, Reinier Hernandez, Charles P. Theuer, Todd E. Barnhart, Weibo Cai

**Affiliations:** 1 Department of Radiology, University of Wisconsin - Madison, WI, USA; 2 Materials Science Program, University of Wisconsin - Madison, WI, USA; 3 Department of Medical Physics, University of Wisconsin - Madison, WI, USA; 4 TRACON Pharmaceuticals, Inc., San Diego, CA, USA; 5 University of Wisconsin Carbone Cancer Center, Madison, WI, USA; 6 These authors contributed equally to this work.; 7 Current address: Departments of Radiology and Medical Physics, University of Wisconsin - Madison, Room 7137, 1111 Highland Avenue, Madison, WI 53705-2275, United States.

## Abstract

Hollow mesoporous silica nanoparticle (HMSN) has recently gained increasing interests due to their tremendous potential as an attractive nano-platform for cancer imaging and therapy. However, possibly due to the lack of efficient *in vivo* targeting strategy and well-developed surface engineering techniques, engineering of HMSN for *in vivo* active tumor targeting, quantitative tumor uptake assessment, multimodality imaging, biodistribution and enhanced drug delivery have not been achieved to date. Here, we report the *in vivo* tumor targeted positron emission tomography (PET)/near-infrared fluorescence (NIRF) dual-modality imaging and enhanced drug delivery of HMSN using a generally applicable surface engineering technique. Systematic *in vitro* and *in vivo* studies have been performed to investigate the stability, tumor targeting efficacy and specificity, biodistribution and drug delivery capability of well-functionalized HMSN nano-conjugates. The highest uptake of TRC105 (which binds to CD105 on tumor neovasculature) conjugated HMSN in the 4T1 murine breast cancer model was ~10%ID/g, 3 times higher than that of the non-targeted group, making surface engineered HMSN a highly attractive drug delivery nano-platform for future cancer theranostics.

Hollow mesoporous silica nanoparticle (HMSN), with a large cavity inside each original mesoporous silica nanoparticle (MSN, a well-known nanocarrier which has been intensively investigated for drug delivery applications since 2000^1^,^2^), has recently gained increasing interests due to their tremendous potential as more advantageous nano-platform for cancer imaging and therapy^3^,^4^. The last several years have witnessed a rapid development in engineering of functionalized HMSNs (*f*-HMSNs) with various types of inorganic functional nanocrystals integrated into the system for imaging and therapeutic applications^5^,^6^,^7^,^8^,^9^. For example, Gd^3+^-doped upconversion nanoparticle encapsulated HMSN has been developed for the integration of optical imaging, magnetic resonance imaging (MRI), chemotherapy and radiotherapy^10^. Another fascinating study is the combination of ultrasound imaging and MRI within one single HMSN, where MRI could potentially be employed as the pre-surgical evaluation tool while ultrasound can be exploited for real-time guidance during high intensity focused ultrasound (HIFU) therapy^11^.

In spite of the progress, most of the existing reports have been focused on the design of sophisticated *f*-HMSNs without fully examining their potential for actively targeted cancer imaging and/or therapy after systemic intravenous (*i.v.*) administration. Currently, very few reports about the quantitative assessment of *in vivo* tumor uptake, biodistribution and clearance studies of HMSN exist in the literature. Applying HMSN to *in vivo* targeted imaging and drug delivery is still considered as one of the major challenges in this field, possibly due to the lack of efficient *in vivo* targeting strategy and well-developed surface engineering techniques.

Recently, we reported the first example of *in vivo* tumor targeting and enhanced drug delivery of TRC105 (a human/murine chimeric IgG1 monoclonal antibody, which binds to both human and murine CD105^12^) antibody conjugated MSN, and demonstrated the promising potential in applying tumor vasculature targeting strategy to functionalized nanoparticles for possible better therapeutic outcome^13^. However, the drug loading capacity of MSN needs to be further improved and the highest tumor uptake was not ideal (~6 percentage injected dose per gram of tissue [%ID/g]).

Here, we report the *in vivo* tumor targeted positron emission tomography (PET)/near-infrared fluorescence (NIRF) dual-modality imaging and enhanced drug delivery of HMSN by using a generally applicable surface engineering method. The anti-cancer drug loading capacity of HMSN was found to be 3–15 times higher than previously reported MSN. By using CD105 (also known as endoglin, which is a validated marker for tumor angiogenesis and almost exclusively expressed on proliferating tumor endothelial cells^14^,^15^) based tumor vasculature targeting strategy, we achieved over 3-fold higher tumor uptake (~10%ID/g at 4 h post-injection [*p.i.*]) in targeted group when compared with the non-targeted group (~3%ID/g at 4 h *p.i.*). Systematic *in vitro* and *in vivo* studies were performed to investigate the stability, targeting efficacy and specificity, biodistribution and drug delivery capability of well-functionalized HMSN nano-conjugates. With the growing interests in using HMSN as a drug delivery nano-platform, this study may pave the way for future cancer theranostics using HMSN, since silica is generally regarded as safe (GRAS) by the Food and Drug Administration (FDA)^4^.

## Results

### Synthesis and characterization

**Figure 1:**
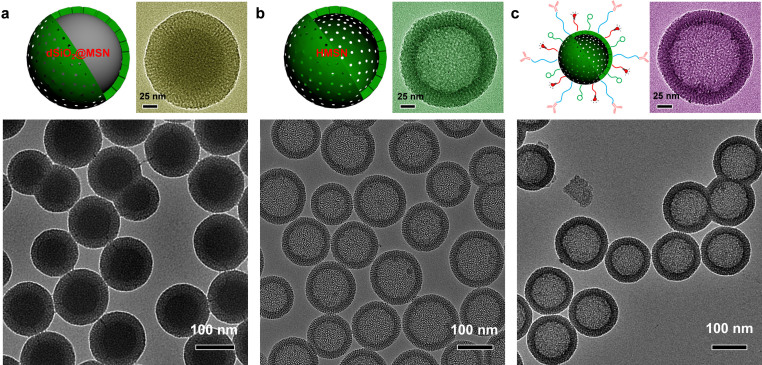
Synthesis of HMSN and HMSN-ZW800-TRC105. (a) TEM image of dSiO_2_@MSN. Upper left & right: a scheme and TEM image of single dSiO_2_@MSN (*dark core represents dSiO_2_ core, while lighter shell is MSN*). (b) TEM image of HMSN. Upper left & right: a scheme and TEM image of single HMSN. (c) TEM image of HMSN-ZW800-TRC105. Upper left & right: a scheme and TEM image of single HMSN-ZW800-TRC105.

Uniform HMSN with an average particle size of 150 nm was synthesized following the literature procedures with improved Na_2_CO_3_-etching process^16^. Generally, three steps were involved. Firstly, uniform ~100 nm sized dense silica (dSiO_2_) nanoparticles were prepared as hard templates using a modified Stöber method^17^. Secondly, shell thickness controllable MSN layer was coated on each dSiO_2_ using cetyltrimethylammonium chloride (*i.e.* CTAC) as the soft templates at 80°C, forming dSiO_2_@MSN(CTAC) (Figure 1a*, dark core represents dSiO_2_ core, while lighter shell is MSN*). Thirdly, as-synthesized dSiO_2_@MSN(CTAC) together with free CTAC (~66.7 mg/mL) in the solution was transferred to 50°C water bath, and kept etching for 30 min after addition of Na_2_CO_3_ (21.2 mg/mL). In comparison with previously reported method^16^, which usually needs more than 10 h of etching, our modified method avoids the tedious washing steps with no re-addition of CTAC, and the etching process could be easily completed within 30 min possibly due to the increased CTAC concentration. Figure 1b shows the representative transmission electron microscopy (TEM) image of as-prepared HMSN(CTAC) with oriented mesoporous shell structures. The surfactant CTAC was later removed *via* an extraction process by stirring HMSN(CTAC) in 1 wt% solution of NaCl in methanol^18^. The pore size of HMSN was 2-3 nm, similar to that of MSN as we reported previously^13^. The diameter of hollow space was ~100 nm, and the thickness of MSN shell was measured to be ~25 nm. Both hollow cavity and MSN shell thickness are readily tunable. For example, using smaller size dSiO_2_ (~50 nm), which could be synthesized in a water-in-oil reverse microemulsion^19^, uniform HMSN with a smaller cavity inside (~50 nm) and thinner MSN shell (~7.5 nm) outside could also be synthesized (Figure S1).

As proposed by Zheng *et al*., selective etching of dSiO_2_ core and redeposition of the soluble silicate species are both important steps during the formation of HMSN^16^. Cationic surfactants (in our case CTAC) play vital roles in such etching-redeposition mechanism^16^. During the etching process, positively charged cetyltrimethylammonium cations (CTA^+^) will first absorb to the surface of dSiO_2_@MSN(CTAC) *via* electronic attraction. Subsequently, with the presence of Na_2_CO_3_ in the solution and CTAC in the MSN shell, selective etching of silica from the dSiO_2_ core starts and will be accelerated by the surrounding free CTAC, forming uniform HMSN(CTAC) after the redeposition process.

We also found that, to selectively etch out the dSiO_2_ core and leave the MSN shell intact, the CTAC concentration, etching temperature and etching time should be tightly-controlled. Only dSiO_2_ will be obtained, and all the MSN shell will be etched away if the concentration of CTAC was kept at below 6.25 mg/mL (Figure S2a). Increasing the CTAC concentration up to 66.7 mg/mL will result in uniform HMSN only when etching is done in a water bath with a mild temperature (*i.e.* 50°C) for a relatively short time (*e.g.* 30–60 min), as shown in Figure S2b, c. Too high etching temperature (*e.g.* 80°C) and too long etching time will easily cause over-etching of HMSN, leaving aggregated nanoparticles with highly rough surfaces (Figure S2d,e,f).

**Figure 2:**
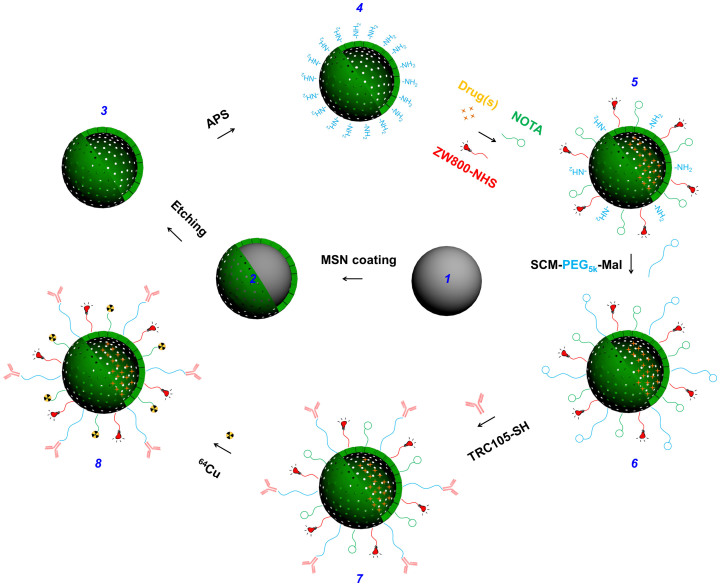
Surface engineering of HMSN. Uniform dense silica (dSiO_2_, ***1***) was firstly synthesized and coated with a shell of MSN, forming dSiO_2_@MSN (***2***). Tightly-controlled Na_2_CO_3_ etching step was introduced to selectively etch dSiO_2_ away, leaving uniform HMSN (***3***). As-synthesized HMSN was then surface modified with (3-Aminopropyl)triethoxysilane (APS) to form amino groups conjugated HMSN-NH_2_ (***4***) before further bio-conjugations. Anti-cancer drugs (*i.e.* DOX) were then loaded, followed by NIR dye (*i.e.* ZW800) and ^64^Cu chelator (*i.e.* NOTA) conjugations, forming NOTA-HMSN(DOX)-ZW800 (***5***). Afterward, nano-conjugate was PEGylated with SCM-PEG_5k_-Mal to render its stability in biological buffers (*e.g.* PBS), forming NOTA-HMSN(DOX)-ZW800-PEG-Mal (***6***). Then, thiolated anti-CD105 antibody (*i.e.* TRC105-SH) was conjugated to the nanoparticle to obtain NOTA-HMSN(DOX)-ZW800-PEG-TRC105 (***7***). Lastly, PET isotope ^64^Cu (*t*_1/2_ = 12.7 h) was used to label the nanoparticle, forming ^64^Cu-NOTA-HMSN(DOX)-ZW800-PEG-TRC105 (***8***).

Surface engineering plays a vital role in *in vivo* applications of nanoparticles, including HMSN. Figure 2 shows the major steps involved in the synthesis of multifunctional HMSN starting from hard template of dSiO_2_ (***1***). After the generation of uniform HMSN (***3***), the nanoparticles were surface modified with amino groups (-NH_2_) to obtain HMSN-NH_2_ (***4***) before further bio-conjugations. Small molecular anti-cancer drugs (*e.g.* doxorubicin hydrochloride [DOX]) could be loaded inside the hollow space, following by near-infrared (NIR) dye (*i.e.* ZW800) and ^64^Cu-chelator (*i.e.* 1,4,7-triazacyclononane-triacetic acid [NOTA]) conjugations, forming NOTA-HMSN(DOX)-ZW800 (***5***). Afterward, the nano-conjugate was PEGylated with SCM-PEG_5k_-Mal (Mal denotes maleimide) to render stability in aqueous biological buffers, forming NOTA-HMSN(DOX)-ZW800-PEG-Mal (***6***). Afterward, thiolated anti-CD105 antibody (*i.e.* TRC105-SH) was conjugated to the nanoparticle to obtain NOTA-HMSN(DOX)-ZW800-PEG-TRC105 (***7***). Finally, PET isotope ^64^Cu with a half-life of 12.7 h was used to label the nanoparticle, forming ^64^Cu-NOTA-HMSN(DOX)-ZW800-PEG-TRC105 (***8***, abbreviated as ^64^Cu-HMSN(DOX)-ZW800-TRC105 for clarity consideration). In comparison with our previously reported surface modification method which starts with thiolated MSN^13^, nano-conjugates with much better stability could be obtained with only one PEGylation step using the current protocol. More importantly, it could be applied to the surface engineering of other silica-related nanoparticles. Since all the HMSN conjugates will contain the same NOTA and PEG chains (5 kDa) that were covalently linked to HMSN, both “NOTA” and “PEG” were omitted from the acronyms of the final conjugates for clarity considerations.

A representative TEM image of HMSN-ZW800-TRC105 is shown in Figure 1c, where no obvious morphology change of HMSN could be observed after multi-step surface modification. The final nano-conjugate could be well-dispersed in phosphate buffered saline (PBS). Average size of HMSN-ZW800-TRC105 was found to be ~150 nm, which is slightly smaller than the *Z-average* size (*i.e.* 194.4 nm) measured by dynamic light scattering (DLS). Figure S3 also presents the size distribution of HMSN-ZW800-TRC105, acquired by DLS. Slightly negatively charged surface (−5.1 ± 0.9 mV) of HMSN-ZW800-TRC105 was also observed.

**Figure 3:**
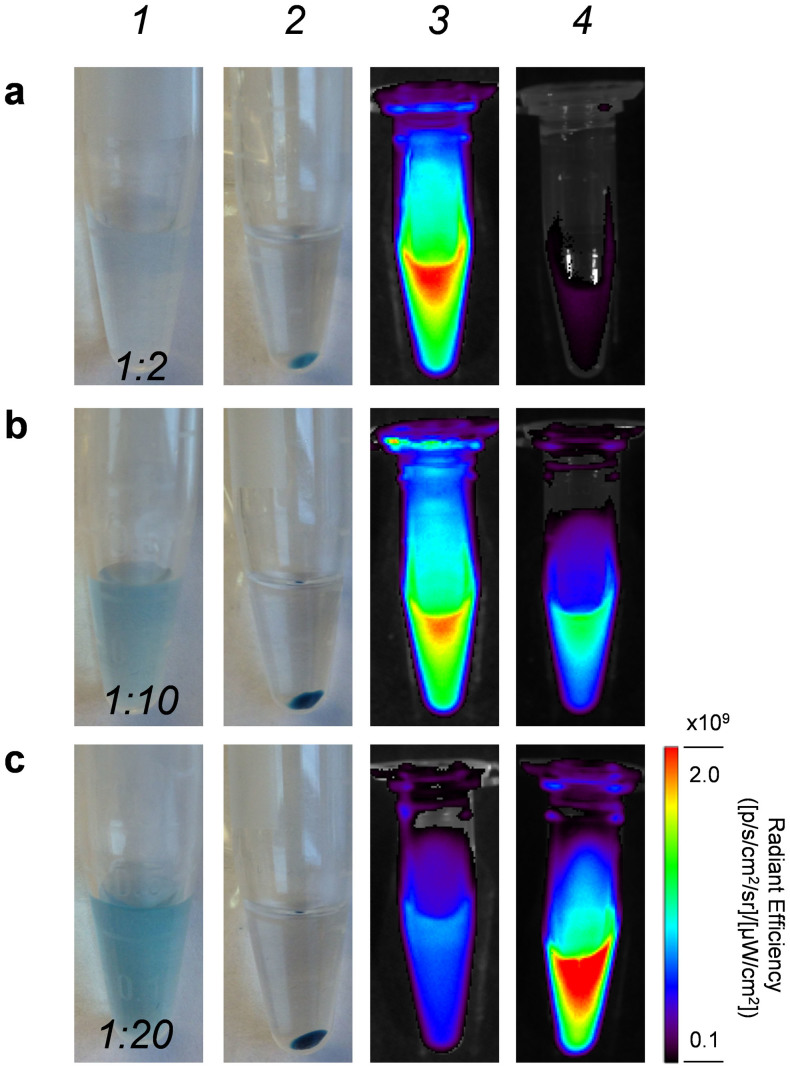
Dye-dye self-quenching study. The molar ratio of HMSN to ZW800 was fixed to be (a) 1:2, (b) 1:10, (c) 1:20. Digital photos of HMSN-ZW800 showing nanoparticles in water before (*Lane 1*) and after centrifugation (*Lane 2*). (*Lane 3*) Optical imaging of HMSN-ZW800 with different amount of dye per nanoparticle. (*Lane 4*) Optical imaging of ZW800 dye in the supernatant after each reaction, indicating unconjugated ZW800. Images were acquired using an IVIS spectrum *in vivo* imaging system (*Ex* = 745 nm, *Em* = 800 nm).

Zwitterionic NIR fluorophore ZW800 (also named as ZW800-1) has recently been demonstrated as a better NIR dye (*Ex* = 745 nm, *Em* = 800 nm) for providing a significantly improved signal-to-background ratio during *in vivo* image-guided surgery, in comparison with traditional dyes such as Cy5.5 and IRDye800CW^20^. Therefore, ZW800-NHS ester was covalently conjugated to the outer surface of HMSN-NH_2_ to render the nanoparticle NIRF imaging capability. To avoid the possible dye-dye self-quenching, the optimal ratio between HMSN and ZW800 was carefully investigated (Figure 3). The lowest degree of ZW800 quenching (~4.8%) was observed when keeping the mole ratio as low as 1:2, where up to 81.4% conjugation efficiency was achieved with each HMSN covered by approximately 1.6 ZW800 dyes (Figure 3a, Table S1). Increasing the amount of dyes while keeping that of HMSN unchanged would increase the number of dye per HMSN to larger than 8, as shown in Table S1, however it also caused severe dye-dye quenching: About 74% and 92% degree of quenching were estimated when fixing the ratio to be 1:10 and 1:20, respectively (Figure 3b,c *and* Table S1). Therefore, the optimized ratio of 1:2 was used in the following *in vivo* tumor targeted imaging studies.

### *In vitro* CD105 targeting of HMSN-ZW800-TRC105

**Figure 4:**
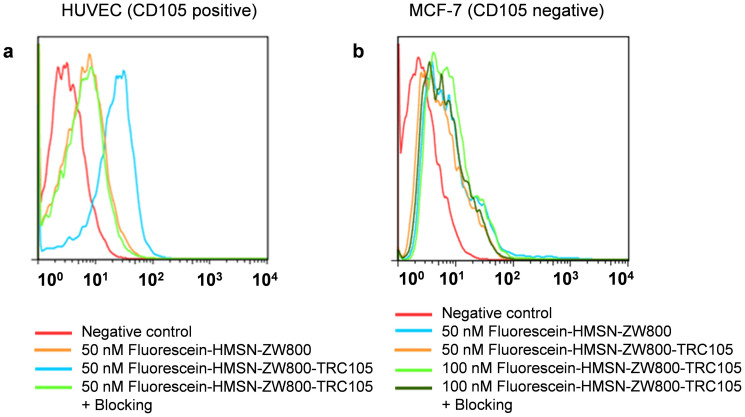
*In vitro* CD105 targeting. Flow cytometry analysis of fluorescein conjugated HMSN-ZW800-TRC105 in (a) HUVEC (CD105 positive) and (b) MCF-7 (CD105 negative) cells after 30 min incubation and subsequent washing. For non-targeted group, same concentration of fluorescein conjugated HMSN-ZW800 was used. In blocking group, free TRC105 (500 μg/mL) was added before adding fluorescein conjugated HMSN-ZW800-TRC105. All incubation time were kept the same as 30 min.

Before the *in vivo* tumor targeted imaging, human umbilical vein endothelial cells (HUVECs, CD105 positive) and MCF-7 human breast cancer cells (CD105 negative) were used for flow cytometry studies to confirm the *in vitro* CD105 targeting efficiency of the HMSN-ZW800-TRC105. Due to the lack of NIR excitation light source in BD FACSCalibur four-color analysis cytometer (equipped with 488 and 633 nm lasers), NHS-fluorescein was conjugated to the surface of nanoparticles to facilitate such investigation. The flow cytometry results from Figure 4a indicated that incubation with fluorescein conjugated HMSN-ZW800-TRC105 (50 nM, targeted group) could significantly enhance the mean fluorescence intensity of HUVECs, while treatment with fluorescein conjugated HMSN-ZW800 (50 nM, non-targeted group), or fluorescein conjugated HMSN-ZW800-TRC105 with a blocking dose of TRC105 (500 μg/mL, blocking group), only gave minimal fluorescence enhancement. In contrast, incubating fluorescein conjugated HMSN-ZW800-TRC105 or fluorescein conjugated HMSN-ZW800 with MCF-7 cells only showed background fluorescence level for all groups (Figure 4b), demonstrating low non-specific binding of functionalized HMSN conjugates in CD105 negative cells. Taken together these findings, successful CD105 targeting of HMSN-ZW800-TRC105 was demonstrated *in vitro*.

### *In vivo* tumor targeting and PET/NIRF dual-modality imaging

PET is highly sensitive, quantitative, clinically relevant, and has excellent tissue penetration of signal^21^,^22^,^23^,^24^. Labeling nanoparticles with positron-emitting radionuclides has been generally recognized as the most accurate means for non-invasive evaluation of their biodistribution and pharmacokinetics^25^,^26^,^27^,^28^. For *in vivo* tumor targeted PET imaging, NOTA-HMSN-ZW800-TRC105 was labeled with ^64^Cu and purified using PD-10 columns with PBS as the mobile phase. The radioactivity fractions (typically elute between 3.0 and 4.0 mL) were collected for further *in vivo* experiments. A typical size exclusion column chromatography profile is provided in Figure S4. Inset in Figure S4 also shows the PET and NIRF imaging of fraction 3.0–3.5 mL, demonstrating the successful synthesis of ^64^Cu-HMSN-ZW800-TRC105.

Since PET imaging detects the radioisotopes (*e.g.* ^64^Cu in this work) rather than the HMSN conjugates *per se*, excellent *in vivo* stability is a prerequisite for PET to truly reflect the *in vivo* distribution of ^64^Cu-HMSN-ZW800-TRC105. Serum stability study (Figure S5) of ^64^Cu-HMSN-ZW800-TRC105 showed that over 99% of ^64^Cu remained intact within ^64^Cu-HMSN-ZW800-TRC105 over a 24 h incubation period in whole mouse serum at 37°C while shaking, which indicates high stability of as-synthesized ^64^Cu-HMSN-ZW800-TRC105.

**Figure 5:**
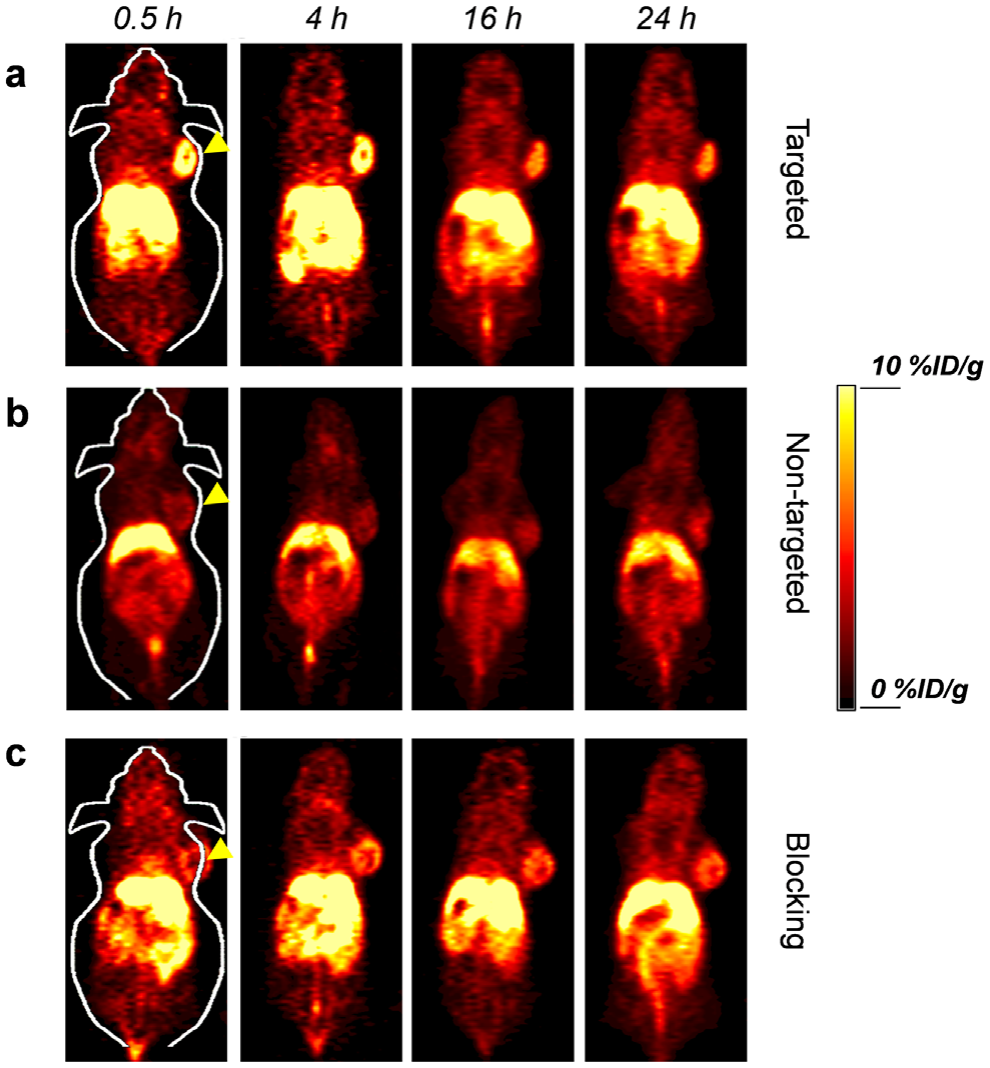
*In vivo* tumor targeted PET imaging. Serial coronal PET images of 4T1 tumor-bearing mice at different time points post-injection of (a) targeted group: ^64^Cu-HMSN-ZW800-TRC105, (b) non-targeted group: ^64^Cu-HMSN-ZW800, or (c) blocking group: ^64^Cu-HMSN-ZW800-TRC105 with a blocking dose (1 mg/mouse) of free TRC105. Tumors were indicated by yellow arrowheads.

*In vivo* tumor targeted PET imaging were then carried out in 4T1 murine breast tumor-bearing mice, which express high level of CD105 on the tumor neovasculature^14^,^15^. Each mouse was injected with 5–10 MBq of ^64^Cu-HMSN-ZW800-TRC105, and time points of 0.5, 4, 16, 24 h *p.i.* were chosen for serial PET scans to show the *in vivo* biodistribution patterns of mice from targeting, non-targeting and blocking groups (Figure 5). Quantitative data obtained from region-of-interest (ROI) analysis of these PET images are also shown in Table S2–S4.

**Figure 6:**
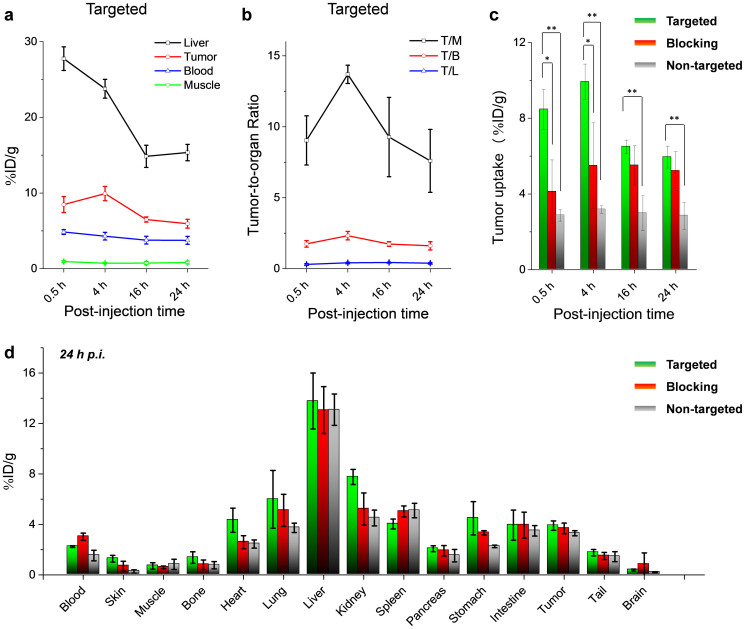
Quantitative ROI analysis and biodistribution studies. (a) Time-activity curve of the liver, 4T1 tumor, blood, and muscle upon *i.v.* injection of ^64^Cu-HMSN-ZW800-TRC105. (b) Time-activity curve of tumor-to-muscle (T/M), tumor-to-blood (T/B) and tumor-to-liver (T/L) ratios. (c) Comparison of 4T1 tumor uptake in ^64^Cu-HMSN-ZW800-TRC105 (targeted group, n = 4), ^64^Cu-HMSN-ZW800 (non-targeted group, n = 4) and the blocking group (n = 3). The difference between 4T1 tumor uptake in targeted group and two control groups were statistically significant (*P < 0.05, **P < 0.01). (d) Biodistribution of 3 groups in 4T1 tumor-bearing mice at the end of the PET scans at 24 h *p.i.*

The accumulation of ^64^Cu-HMSN-ZW800-TRC105 in the 4T1 tumor was found to be 8.5 ± 1.1%ID/g at 0.5 h *p.i.*, and peaked at 9.9 ± 0.9%ID/g at 4 h *p.i.*, as shown in Figure 5a*,*6a*,*S6 and Table S2 (n = 4). In contrast, without the conjugation of TRC105 (*i.e.* passive targeting alone), the 4T1 tumor uptake of ^64^Cu-HMSN-ZW800 was found to be only one third of the targeted group at all of the time points examined (n = 4; Figure 5b*, *Table S3), indicating that TRC105 conjugation could be the controlling factor for enhanced tumor accumulation of ^64^Cu-HMSN-ZW800-TRC105. To further confirm CD105 targeting specificity of ^64^Cu-HMSN-ZW800-TRC105 *in vivo*, blocking studies were also performed. Administration of a blocking dose (1 mg/mouse) of free TRC105 at 1 h before ^64^Cu-HMSN-ZW800-TRC105 injection could significantly reduce the tumor uptake to 4.1 ± 1.7 and 5.5 ± 2.2%ID/g at 0.5 and 4 h *p.i.*, respectively (n = 3, Figure 5c*, *Table S4), clearly demonstrating CD105 specificity of ^64^Cu-HMSN-ZW800-TRC105 *in vivo*. Figure 6c also summarizes the comparison of 4T1 tumor uptake of 3 groups at different time points, where ^64^Cu-HMSN-ZW800-TRC105 shows the highest tumor uptake throughout the study period.

Besides significantly improved tumor targeting efficacy, the tumor-to-muscle (T/M) ratios increased as well. As shown in Figure 6b, T/M value of the targeted group was estimated to be 9.0 ± 1.7 at 0.5 h *p.i.*, and increased up to 13.7 ± 0.6 at 4 h *p.i.*, which is significantly higher than that of non-targeting and blocking groups (Figure S7, Table S6–7). Similar as what we have observed previously with ^64^Cu-MSN-TRC105^13^, besides tumor accumulation, most of ^64^Cu-HMSN-ZW800-TRC105 nano-conjugates were taken up by the reticuloendothelial system (RES) with liver uptake found to be 27.8 ± 1.6%ID/g at 0.5 h *p.i.* and decreased gradually to 15.4 ± 1.1%ID/g at 24 h *p.i.* (n = 4; Figure 6a, Table S2), which was validated by the *ex vivo* biodistribution study (Figure 6d).

Engineering of HMSN to integrate two or more imaging modalities into one nanosystem has become a recent trend, which could provide complementary and more accurate information about the pharmacokinetics of HMSN *in vivo*^8^. Generally speaking, optical imaging is inexpensive, widely available, and highly sensitive for superficial tissues, which has been extensively used for monitoring various molecular/biological events in cells and small animal models^29^,^30^. Although various strategies (*e.g.* organic dye doping, fluorescent nanoparticle encapsulation, *etc.*) have been developed to engineer MSNs for optical imaging applications *in vitro* and *in vivo*^31^, very few reports about the engineering of HMSN for *in vivo* targeted optical imaging exist in the literature.

**Figure 7:**
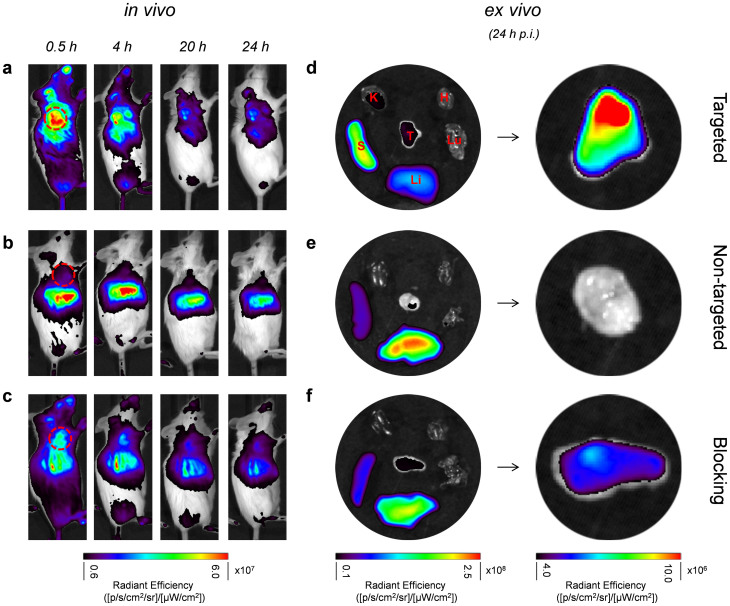
*In vivo* tumor targeted NIRF imaging. (a) Targeted group. (b) Non-targeted group. (c) Blocking group. Tumors were marked by dashed red circles. *Ex vivo* NIRF imaging of major organs (*left*) and tumor-only (*right*) from 4T1 tumor-bearing mice at 24 h *p.i.* (d) Targeted group. (e) Non-targeted group. (f) Blocking group. Kidney (K), spleen (S), liver (Li), lung (Lu), heart (H), tumor (T).

With the presence of optimized number of ZW800 on the surface of each HMSN nano-conjugate, the *in vivo* NIRF imaging was also carried out. Each 4T1 tumor-bearing mouse was first shaved and injected with HMSN-ZW800-TRC105 with 800 pmol of ZW800. Similar time points (*i.e.* 0.5, 4, 20, 24 h *p.i.*) as that of PET imaging studies were chosen for serial optical imaging using the IVIS Spectrum system (*Ex* = 745 nm, *Em* = 800 nm). Figure 7 shows the *in vivo* NIRF images of mice from 3 groups. Significantly higher optical intensity of tumor (marked with red circle) from targeted group could be clearly seen (Figure 7a–c), and was confirmed by the *ex vivo* imaging (Figure 7d–f*, right lane*). For all 3 groups, tumor signals decreased over time, possibly due to the wash-out of nano-conjugates *in vivo*. *Ex vivo* imaging of major organs, *e.g.* liver (Li), spleen (S), lung (Lu), kidney (K), heart (H) and tumor (T), showed similar distribution patterns as what has been shown in Figure 6d, with liver and spleen being the major nanoparticle uptake organs.

### Enhanced *in vivo* tumor targeted drug delivery

Although MSN has been considered as a drug delivery nano-platform for over 10 years^1^, it was not until recently that the first example of quantitative assessment of tumor targeting efficacy and *in vivo* enhanced drug delivery using TRC105 conjugated MSN(DOX) was demonstrated by our group^13^. In this work, we demonstrated that HMSN could become a more attractive nanocarrier for future drug delivery applications.

**Figure 8:**
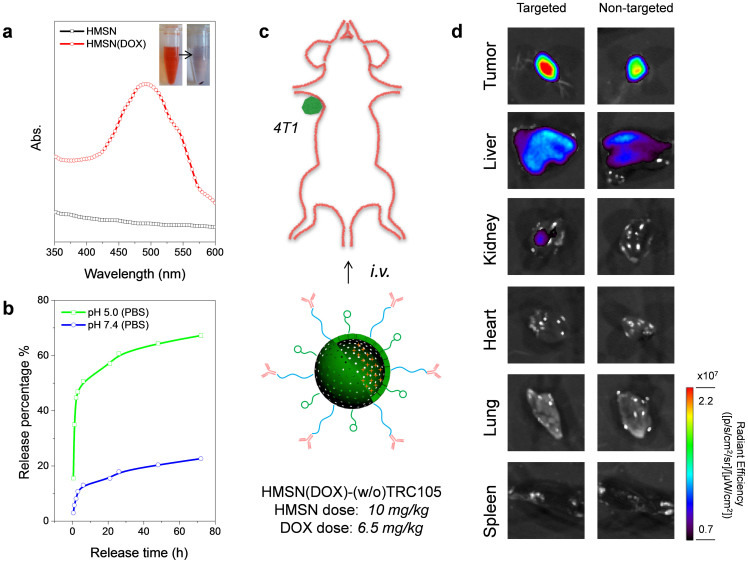
*In vivo* enhanced drug delivery. (a) UV-vis spectra of HMSN and HMSN(DOX) in aqueous solution. (b) pH-sensitive release profiles of HMSN(DOX) at pH 5.0 and pH 7.4 in PBS. (c) A schematic illustration showing the in vivo drug delivery after i.v. injection of HMSN(DOX)-(w/o)-TRC105 in 4T1 tumor-bearing mouse, created by F.C. (d) *Ex vivo* optical imaging of major organs after *i.v.* injection of HMSN(DOX)-(w/o)-TRC105 in 4T1 tumor-bearing mice. HMSN dose was 10 mg/kg, while the DOX dose was 6.5 mg/kg (*Ex* = 465 nm, *Em* = 580 nm).

In comparison with the DOX loading capacity in MSN (*i.e.* 71.6–481.6 mg/g), with the presence of hollow cavity inside HMSN, a significantly improved (about 3–15 times higher) drug loading capacity, up to 1129.2 mg/g, was achieved (Figure S8a). The successful loading of DOX was further supported by the UV-vis absorbance spectra of HMSN(DOX), which exhibited the characteristic absorption peak at around 480 nm (Figure 8a*, red line*). DOX is known to be intrinsically pH sensitive^32^. Due to the protonation of silanols (-Si-OH) in HMSN with the decrease of pH, electrostatic interaction between DOX and HMSN will be decreased, causing the dissociation of DOX from the silica surface and mesoporous channels. A pH-sensitive DOX release profile was also observed in HMSN(DOX), where shows a faster DOX release rate at acidic condition (pH 5.0) than that at neutral condition (pH 7.4, Figure 8b). Besides loading one type of hydrophilic anti-cancer drug, *e.g.* DOX, we also demonstrated here that HMSN could be a promising nanocarrier for loading certain hydrophobic drugs, such as Sunitilib (SUN, Figure S9), as well as double-loading with both SUN and DOX (Figure S10).

As a proof-of-concept, we further demonstrated the feasibility of enhanced tumor targeted drug delivery *in vivo* using TRC105 conjugated HMSN(DOX), denoted as HMSN(DOX)-TRC105, after *i.v.* injection in 4T1 tumor-bearing mice (HMSN dose: 10 mg/kg, DOX dose: 6.5 mg/kg). Half an hour after injection of HMSN(DOX)-(w/o)-TRC105 (w/o denotes with and without), the major organs were collected and imaged in the IVIS system (*Ex* = 465 nm; *Em* = 580 nm) to detect the presence of DOX, as shown in Figure 8c, d. It is important to note that due to different absorption/scattering behaviors of DOX in various tissues, optical signal intensities from different organs may not accurately reflect the absolute uptake level of injected HMSN(DOX)-(w/o)-TRC105. For example, although liver is the dominant organ for HMSN nano-conjugates accumulation, as evidenced in our PET/NIRF imaging and biodistribution studies (Figure 5, 6d, and 7), only weak optical signal could be observed based on *ex vivo* optical imaging because of its dark color and strong absorbance of visible DOX fluorescence (Figure 8d). In contrast, due to the much lighter color of tumor tissue, dominant optical signal from DOX could be observed in mice injected with HMSN(DOX)-TRC105, which is significantly stronger than the control group without TRC105 conjugation. Therefore, the significantly enhanced tumor targeting efficiency and drug (or multiple drugs) loading capacity will make antibody conjugated HMSN a highly attractive nano-platform for future cancer targeted imaging and therapy.

## Discussion

Although molecular imaging of functionalized HMSN using imaging techniques, such as MRI^11^, optical imaging^10^ and ultrasound^7^, has been reported previously^8^, to date most of the studies have been primarily focused on optimization of nanoparticle synthesis. To the best of our knowledge, no *in vivo* tumor active-targeted PET imaging, quantitative tumor uptake, and biodistribution studies of HMSN have been reported. By surface engineering of uniform HMSN with radioisotopes (*i.e.* ^64^Cu), NIR dyes (*i.e.* ZW800) and CD105 targeting antibodies (*i.e.* TRC105), we reported here the first example of *in vivo* tumor vasculature targeted PET/NIRF dual-modal imaging of ^64^Cu-HMSN-ZW800-TRC105. An average of 3-fold enhancement of tumor accumulation was achieved in targeted group when compared with the non-targeted group. The CD105 specific targeting was further confirmed with blocking studies both *in vitro* and *in vivo*. It is also worthy to mention that the highest 4T1 tumor uptake of ^64^Cu-HMSN-ZW800-TRC105 (9.9 ± 0.9%ID/g at 4 h *p.i.*, n = 4) was nearly 2 times higher than our previously reported ^64^Cu-MSN-TRC105 (5.9 ± 0.4%ID/g at 5 h *p.i.*, n = 3)^13^, as shown in Figure S8b, resulting in remarkable enhancement in targeting efficacy as well as T/M ratio (13.7 ± 0.6 at 4 h *p.i.* vs 6.2 ± 1.8 at 5 h *p.i.*)^13^.

Meanwhile, although tumor vasculature targeting has recently been demonstrated to be a versatile strategy for many well-functionalized nanomaterials regardless of tumor type^28^, the absolute level of tumor accumulation for most of the nanomaterials investigated to date is still relatively low, typically in the range of 3–6%ID/g^13^,^25^,^33^,^34^,^35^,^36^,^37^, significantly lower than that of liver uptake (usually 20–30%ID/g or higher for most of the nanomaterials upon *i.v.* injection). To the best of our knowledge, ^64^Cu-HMSN-ZW800-TRC105 is one of the very few vasculature targeted nanoparticles (another sample is RGD peptide conjugated single-walled carbon nanotubes which target integrin α_v_β_3_^38^) that could have about 10%ID/g of *in vivo* tumor uptake. Although we believe surface engineering of nanoparticles could play a vital role, elucidating the exact reasons behind such remarkably enhanced tumor uptake requires systematic side-by-side tumor uptake comparison of well-engineered HMSN with varied particle size, morphology, surface charge, antibody density, *etc*., which deserves comprehensive investigation in the future. Considering the presence of large drug reservoir (*i.e.* hollow space) inside each HMSN, TRC105 conjugated HMSN might become a highly attractive drug delivery platform for future cancer theranostics.

In conclusion, we reported the engineering of uniform HMSN for remarkably enhanced *in vivo* tumor vasculature targeted PET/NIRF dual-modality imaging and enhanced drug delivery. Uniform and size-controllable HMSN was synthesized with a modified hard-templating method, which were subjected to a generally applicable surface engineering process, including amino groups functionalization, anti-cancer drug loading, NIR dye and NOTA linkages, PEGylation, antibody conjugation and radiolabeling, forming ~150 nm sized mono-disperse ^64^Cu-HMSN(DOX)-ZW800-TRC105 nano-conjugates. The optimal ratio between HMSN and ZW800 was also carefully investigated to avoid the self-quenching effect, and found to be 1:2 with less than 5% of quenching. Systematic *in vitro* and *in vivo* studies was performed to investigate the stability, targeting efficacy and specificity, biodistribution and drug delivery capability of well-modified HMSN nano-conjugates. Quantitative PET imaging study showed an over 3-fold higher of tumor uptake in the targeted group when compared with the non-targeted group, demonstrating that ^64^Cu-HMSN-ZW800-TRC105 is one of the very few vasculature targeting nanoparticles with high (~10%ID/g) tumor uptake. Furthermore, with the presence of hollow space inside HMSN, 3–15 times higher of DOX loading capacity has also been achieved when compared with MSN, with the highest loading capacity estimated to be 1129.2 mg/g. *In vivo* enhanced DOX delivery was also demonstrated in 4T1 tumor-bearing mice. Take together these results, active tumor targeting and drug delivery of functionalized HMSN may pave the way for further *in vivo* targeted cancer therapeutic studies as well as potential clinical translational of this promising type of nano-platform, since silica is generally regarded as safe by the FDA.

## Methods

### Synthesis of uniform HMSN

Uniform HMSN with an average particle size of 150 nm was synthesized following the literature procedure with improved Na_2_CO_3_-etching process^16^. Three major steps were involved in the synthesis of HMSN. *Firstly*, synthesis of uniform ~100 nm sized dSiO_2_ using a modified Stöber method^17^. In a typical synthesis, 35.7 mL of absolute ethanol was mixed with 5 mL water and 0.8 mL of ammonia and stirred for 5–10 minutes at room temperature. Then 1 mL of TEOS was added and the mixture was allowed to react at room temperature for 1 h. Afterward, dSiO_2_ nanoparticles was washed with water and ethanol and suspended in 20 mL of water.

*Secondly*, synthesis of dSiO_2_@MSN. CTAC (2 g) and TEA (20 mg) were dissolved in 20 mL of high Q water and stirred at room temperature for 1 h. Then, 10 mL of dSiO_2_ water solution was added and stirred at room temperature for 1 h before addition of 0.15 mL of TEOS. The mixture was stirred for 1 h at 80°C in a water bath to form dSiO_2_@MSN.

*Thirdly*, etching of dSiO_2_@MSN to form HMSN. Both mixture and water bath were cooled down to 50°C followed by addition of 636 mg of sodium carbonate (Na_2_CO_3_), which was under constant stirring for 30 min to form HMSN. To remove the CTAC, the product was extracted for 24 h with a 1 wt% solution of NaCl in methanol at room temperature. This process was carried out for at least 3 times to ensure complete removal of CTAC.

### Synthesis of HMSN-NH_2_

To functionalize the HMSN surface with –NH_2_ groups, as-synthesized HMSN was first dispersed in 20 mL of absolute ethanol, followed by addition of 1 mL of APS. The system was sealed and kept at 86–90°C in a water bath for 24 h. Afterward, the mixture was centrifuged and washed with ethanol for several times to remove the residual APS. The HMSN-NH_2_ could be well-dispersed in water, and the concentration of –NH_2_ groups (nmol/mL) was measured using a Kaiser test kit.

### Synthesis of NOTA-HMSN-ZW800-PEG-TRC105

To conjugated HMSN with ZW800, 2 nmol of ZW800 was mixed with HMSN-NH_2_ (with ~100 nmol of -NH_2_ groups), and reacted for 2 h at room temperature (pH 8.5–9.0) to form HMSN-ZW800. Then, p-SCN-Bn-NOTA (~53 nmol) in dimethyl sulfoxide was allowed to react with HMSN-NH_2_ at pH 8.5 to obtain NOTA-HMSN-ZW800. Afterward, 2 mg (400 nmol) of SCM-PEG_5k_-Mal was added and reacted for another 1 h, resulting in NOTA-HMSN-ZW800-PEG-Mal. NOTA-HMSN-ZW800-PEG-TRC105 could be obtained by reacting TRC105-SH (2.5 nmol, 50 nmol/mL) with NOTA-HMSN-ZW800-PEG-Mal (0.5 nmol, 2 nmol/mL).

### ^64^Cu labeling and serum stability studies

^64^CuCl_2_ (~148 MBq) was diluted in 300 μL of 0.1 M sodium acetate buffer (pH 6.5) and added to NOTA-HMSN-ZW800-PEG-TRC105 or NOTA-HMSN-ZW800-PEG. The reaction was allowed to proceed at 37°C for 30 min with constant shaking. ^64^Cu-NOTA-HMSN-ZW800-PEG-TRC105 and ^64^Cu-NOTA-HMSN-ZW800-PEG were purified using PD-10 columns with PBS as the mobile phase. The radioactivity fractions (typically between 3.0 and 4.0 mL, total of 1 mL) were collected for further *in vivo* PET imaging experiments. After 6 mL of PBS, the unreacted ^64^Cu may start to elute from the column. The whole procedure of ^64^Cu labeling and purification of the HMSN nano-conjugates could be completed within 60 min.

For serum stability studies, ^64^Cu-HMSN-ZW800-TRC105 was incubated in whole mouse serum at 37°C for up to 24 h (the time period investigated for serial PET imaging, which is about two half-lives of ^64^Cu). Portions of the mixture were sampled at different time points and filtered through 10 kDa cutoff filters. The filtrates were collected, and the radioactivity was measured. The percentages of retained (*i.e.* intact) ^64^Cu on the HMSN-PEG conjugates (^64^Cu-HMSN-ZW800-TRC105) were calculated using the equation [(total radioactivity - radioactivity in filtrate)/total radioactivity] × 100%.

### PET/NIRF imaging and biodistribution studies

All animal studies were conducted under a protocol approved by the University of Wisconsin Institutional Animal Care and Use Committee. PET and PET/CT scans at various time points *p.i.* using a microPET/microCT Inveon rodent model scanner (Siemens Medical SolutionsUSA, Inc.), image reconstruction, and ROI analysis of the PET data were performed similar as described previously^34^,^35^,^39^,^40^,^41^,^42^. Quantitative PET data were presented as %ID/g. Tumor-bearing mice were each injected with 5–10 MBq of ^64^Cu-HMSN-ZW800-TRC105 or ^64^Cu-HMSN-ZW800 *via* tail vein before serial PET scans. Another group of three 4T1 tumor-bearing mice were each injected with 1 mg of unlabeled TRC105 at 1 h before ^64^Cu-HMSN-ZW800-TRC105 administration to evaluate the CD105 targeting specificity of ^64^Cu-HMSN-ZW800-TRC105 *in vivo* (*i.e.* blocking experiment).

After the last PET scans at 24 h *p.i.*, biodistribution studies were carried out to confirm that the %ID/g values based on PET imaging accurately represented the radioactivity distribution in tumor-bearing mice. The radioactivity in the tissue was measured using a gamma-counter (Perkin-Elmer) and presented as %ID/g (mean ± SD).

For *in vivo* NIRF imaging, each 4T1 tumor-bearing mouse was injected with 0.5 nmol of HMSN-ZW800-TRC105 (targeted group) with the amount of ZW800 estimated to be ~800 pmol. The mouse was then imaged using an IVIS Imaging System (*Ex* = 745 nm, *Em* = 800 nm) at different time points. At 24 h *p.i.*, mouse was sacrificed for *ex vivo* NIRF imaging. For non-targeting and blocking groups, mice were injected with the same amount of HMSN-ZW800 and HMSN-ZW800-TRC105 (together with 1 mg blocking dose of free TRC105), respectively.

### *In vivo* enhanced drug delivery

HMSN(DOX)-TRC105 was first prepared. Typically, 0.4 mg of HMSN-NH_2_ was first loaded with DOX, and then went through same PEGylation and TRC105 conjugation, as described previously. For *in vivo* enhanced drug delivery study, 4T1 tumor bearing mice were *i.v.* injected with HMSN(DOX)-TRC105 (targeted group: 10 mg HMSN/kg, 6.5 mg DOX/kg), and HMSN(DOX) (non-targeted group: 10 mg HMSN/kg, 6.5 mg DOX/kg). The mice were than sacrificed at 0.5 h *p.i.* for *ex vivo* optical imaging in the IVIS system (*Ex* = 465 nm, *Em* = 580 nm).

## Author Contributions

F.C., H.H. and W.C. conceived of the study and wrote the manuscript. F.C., H.H., S.S., S.G., H.F.V., R.H. and T.E.B. performed the experiments. C.P.T. provided TRC105 for the study. All authors reviewed and approved the manuscript.

## Supplementary Material

Supplementary Information SI
