# Supplementary material for: Engineering of Hollow Mesoporous Silica Nanoparticles for Remarkably Enhanced Tumor Active Targeting Efficacy
Source: Sci Rep. 2014 May 30;4:5080. doi: 10.1038/srep05080 (PMC4038837; doi:10.1038/srep05080)
Supplement: Supplementary Information — SI [file srep05080-s1.doc]

**Supporting Information**

**Engineering of Hollow Mesoporous Silica Nanoparticles for Remarkably Enhanced Tumor Active Targeting Efficacy**

***Feng Chen****,^1,6^* ***Hao Hong****,^1,6^ Sixiang Shi,^2^ Shreya Goel,^2^ Hector F. Valdovinos,^3^ Reinier Hernandez,^3^ Charles P. Theuer,^4^ Todd E. Barnhart,^3^ Weibo Cai*^1,2,3,5^*

*^1^ Department of Radiology, University of Wisconsin - Madison, WI, USA*

*^2^ Materials Science Program, University of Wisconsin - Madison, WI, USA*

*^3^ Department of Medical Physics, University of Wisconsin - Madison, WI, USA*

*^4^ TRACON Pharmaceuticals, Inc., San Diego, CA, USA*

*^5^ University of Wisconsin Carbone Cancer Center, Madison, WI, USA*

*^6^ Feng Chen and Hao Hong contributed equally to this work*

**Corresponding Author**

[**wcai@uwhealth.org**](mailto:WCai@uwhealth.org)

Present Addresses

Departments of Radiology and Medical Physics, University of Wisconsin - Madison, Room 7137, 1111 Highland Avenue, Madison, WI 53705-2275, United States.

**Supplementary Figures and Tables**

**Figure S1**. **Synthesis of ~65 nm sized HMSN**. (a) TEM image of ~50 nm sized dSiO_2_. (b) TEM image of ~65 nm sized HMSN.

**Figure S2**. **Optimization of the etching process**. TEM image of HMSN synthesized at conditions of (**a**) 50 ºC etching for 14 h with CTAC concentration of 6.25 mg/mL. (**b**) 50 ºC etching for 30 min with CTAC concentration of 66.7 mg/mL. (**c**) 50 ºC etching for 60 min with CTAC concentration of 66.7 mg/mL. (**d**) 80 ºC etching for 60 min with CTAC concentration of 66.7 mg/mL. (**e**) 80 ºC etching for 30 min with CTAC concentration of 66.7 mg/mL. (**f**) 80 ºC etching for 10 min with CTAC concentration of 66.7 mg/mL. The concentration of Na_2_CO_3_ was 21.2 mg/mL in all cases.

**Figure S3**. **Size distribution study**. DLS size distribution of HMSN-ZW800-TRC105 before ^64^Cu labeling. The Z-average size was measured to be 194.4 nm, and zeta potential was -5.1 ± 0.9 mV.

**Table S1. Dye-dye quenching effect of ZW800 after conjugation to HMSN.**

| ***HMSN:ZW800***  ***ratio*** | ***HMSN***  ***(nmol)*** | ***ZW800 used***  ***(nmol)*** | ***ZW800 in the supernatant***  ***(nmol)*** | ***^[a]^ZW800 in HMSN*** | ***^[b]^Equal amount of ZW800*** | ***^[c]^Conjugation efficacy*** | ***^[d]^Number of ZW800 per HMSN*** | ***^[e]^Degree of quenching*** |
| --- | --- | --- | --- | --- | --- | --- | --- | --- |
| *1:2* | 0.5 | 1.0 | 0.18 | 0.82 | 0.78 | 81.4 % | *1.6* | *4.8 %* |
| *1:10* | 0.5 | 5.0 | 0.89 | 4.11 | 1.07 | 82.2 % | *8.2* | *74 %* |
| *1:20* | 0.5 | 10.0 | 3.13 | 6.87 | 0.57 | 68.7 % | *13.7* | *92 %* |

^[a]^ ZW800 in HMSN = ZW800 used – ZW800 in the supernatant.

^[b]^ Equal amount of ZW800 was calculated by using a linear fitting equation of free ZW800 dyes.

^[c]^ Conjugation efficiency = ZW800 in HMSN/ZW800 used.

^[d]^ Number of dyes per HMSN = ZW800 in HMSN/HMSN.

^[e]^ Degree of quenching = 1-(Equal amount of ZW800/ZW800 in HMSN).

**Figure S4.** **Purification of ^64^Cu-HMSN-ZW800-TRC105**. A size-exclusion column chromatography elution profile for the purification of ^64^Cu-HMSN-ZW800-TRC105. The unreacted ^64^Cu elutes after 6 mL. Inset left: PET imaging of fraction 3.0-3.5 mL; Inset right: NIRF imaging of the same fraction (*Ex*=745 nm, *Em*=800 nm).

**Figure S5**. **Serum stability study of ^64^Cu-HMSN-ZW800-TRC105**. Sample was incubated in complete mouse serum at 37 ºC for 24 h under constant shaking (*550 rpm*).

**Figure S6**. ***In vivo* PET/CT images**. Representative (**a**) PET/CT and (**b**) coronal, (**c**) sagittal and (d) axial PET images of the same mouse from targeted group at 4 h p.i. Tumors were marked with yellow arrows.

**Table S2.** Quantitative PET data of ^64^Cu-HMSN-ZW800-TRC105 (targeted group) based on ROI analysis.

| **Targeted group**  **(n = 4)** | 0.5 h  (%ID/g) | 4 h  (%ID/g) | 16 h  (%ID/g) | 24 h  (%ID/g) |
| --- | --- | --- | --- | --- |
| Liver | 27.8 ± 1.6 | 23.8 ± 1.2 | 14.9 ± 1.5 | 15.4 ± 1.1 |
| Tumor | 8.5 ± 1.1 | 9.9 ± 0.9 | 6.5 ± 0.3 | 6.0 ± 0.6 |
| Blood | 4.9 ± 0.3 | 4.3 ± 0.5 | 3.8 ± 0.5 | 3.8 ± 0.5 |
| Muscle | 0.9 ± 0.1 | 0.7 ± 0.2 | 0.7 ± 0.2 | 0.8 ± 0.2 |

**Table S3.** Quantitative PET data of ^64^Cu-HMSN-ZW800 (non-targeted group) based on ROI analysis.

| **Non-targeted group**  **(n = 4)** | 0.5 h  (%ID/g) | 4 h  (%ID/g) | 16 h  (%ID/g) | 24 h  (%ID/g) |
| --- | --- | --- | --- | --- |
| Liver | 18.7 ± 1.2 | 14.5 ± 0.3 | 10.7 ± 1.0 | 11.1 ± 0.5 |
| Tumor | 2.9 ± 0.3 | 3.2 ± 0.2 | 3.0 ± 0.9 | 2.9 ± 0.7 |
| Blood | 2.3 ± 0.1 | 2.4 ± 0.3 | 2.7 ± 0.4 | 2.8 ± 0.2 |
| Muscle | 0.4 ± 0.1 | 0.4 ± 0.1 | 0.5 ± 0.2 | 0.6 ± 0.2 |

**Table S4.** Quantitative PET data of ^64^Cu-HMSN-ZW800-TRC105 with a blocking dose of TRC105 (1 mg/mouse, blocking group) based on ROI analysis.

| **Blocking group**  **(n = 3)** | 0.5 h  (%ID/g) | 4 h  (%ID/g) | 16 h  (%ID/g) | 24 h  (%ID/g) |
| --- | --- | --- | --- | --- |
| Liver | 28.5 ± 2.9 | 21.2 ± 4.5 | 15.5 ± 2.5 | 16.2 ± 1.7 |
| Tumor | 4.1 ± 1.7 | 5.5 ± 2.2 | 5.5 ± 1.0 | 5.2 ± 1.0 |
| Blood | 5.7 ± 3.1 | 4.0 ± 1.0 | 3.9 ± 0.7 | 4.0 ± 0.6 |
| Muscle | 0.7 ± 0.2 | 0.6 ± 0.1 | 0.7 ± 0.3 | 0.7 ± 0.3 |

**Table S5.** Summary of tumor-to-organ ratios in the targeted group.

| **Targeted group**  **(n = 4)** | 0.5 h | 4 h | 16 h | 24 h |
| --- | --- | --- | --- | --- |
| *T/M* | 9.0 ± 1.7 | 13.7 ± 0.6 | 9.3 ± 2.8 | 7.6 ± 2.2 |
| *T/B* | 1.8 ± 0.2 | 2.3 ± 0.3 | 1.7 ± 0.2 | 1.6 ± 0.3 |
| *T/L* | 0.3 ± 0.1 | 0.4 ± 0.1 | 0.4 ± 0.0 | 0.4 ± 0.0 |

**Table S6.** Summary of tumor-to-organ ratios in the non-targeted group.

| **Non-targeted group**  **(n = 4)** | 0.5 h | 4 h | 16 h | 24 h |
| --- | --- | --- | --- | --- |
| *T/M* | 7.0 ± 2.0 | 8.5 ± 1.7 | 6.7 ± 1.7 | 5.6 ± 2.3 |
| *T/B* | 1.2 ± 0.2 | 1.3 ± 0.2 | 1.1 ± 0.4 | 1.0 ± 0.2 |
| *T/L* | 0.2 ± 0.0 | 0.2 ± 0.1 | 0.3 ± 0.1 | 0.3 ± 0.1 |

**Table S7.** Summary of tumor-to-organ ratios in the blocking group.

| **Blocking group**  **(n = 3)** | 0.5 h | 4 h | 16 h | 24 h |
| --- | --- | --- | --- | --- |
| T/M | 5.5 ± 0.5 | 8.1 ± 2.6 | 7.9 ± 2.0 | 7.7 ± 2.1 |
| T/B | 0.8 ± 0.2 | 1.3 ± 0.3 | 1.4 ± 0.2 | 1.3 ± 0.1 |
| T/L | 0.1 ± 0.0 | 0.3 ± 0.1 | 0.4 ± 0.0 | 0.3 ± 0.0 |

**Figure S7**. **Tumor-to-organ ratios analysis**. (**a**) Targeted group. (**b**) Non-targeted group. (**c**) Blocking groups. For blocking group, 1 mg free TRC105 was injected in each 4T1 tumor-bearing mouse at 1 h before administration of ^64^Cu-HMSN-ZW800-TRC105. T/M: tumor-to-muscle. T/B: tumor-to-blood. T/L: tumor-to-liver.

**Figure S8**. **Drug loading capacity and tumor targeting efficacy comparison between MSN and HMSN.** (**a**) Comparison of DOX loading capacity for HMSN and MSN. (**b**) *In vivo* tumor targeting efficacy of ^64^Cu-HMSN(150nm)-ZW800-TRC105 and ^64^Cu-MSN(80nm)-TRC105.

**Figure S9**. **Loading capacity study of a hydrophobic drug, Sunitinib (SUN)**. UV-vis spectra of HMSN and HMSN(SUN) in water. Inset shows the molecular structure of SUN and HMSN(SUN) before and after centrifugation.

**Figure S10. Hydrophobic/hydrophilic double-drug loading study.** (**a**) Digital photos of HMSN(SUN) after the first round loading with SUN. (**b**) Digital photos of HMSN(SUN+DOX) after the second round loading with DOX. (**c**) UV-vis spectra of HMSN, HMSN(SUN), HMSN(DOX) and HMSN(SUN+DOX).

**Supplementary Methods**

**Materials**. TRC105 was provided by TRACON Pharmaceuticals Inc. (San Diego, CA). PD-10 columns were purchased from GE Healthcare (Piscataway, NJ). Absolute ethanol, cyclohexane, sodium chloride (NaCl), Doxorubicin hydrochloride (DOX) were purchased from Fisher Scientific. ZW800 NHS ester was purchased from The FLARE^TM^ Foundation. SCM-PEG_5k_-Mal was obtained from Creative PEGworks. p-SCN-Bn-NOTA was acquired from Macrocyclics, Inc. (Dallas, TX). NHS-Fluorescein, Chelex 100 resin (50-100 mesh), tetraethyl orthosilicate (TEOS), ammonia (NH_3_^.^H_2_O), Igepal CO-520 (NP-5), triethylamine (TEA), (3-Aminopropyl)triethoxysilane (APS), dimethyl sulfoxide, cetyltrimethylammonium chloride (CTAC, 25 wt%) and Kaiser test kit were purchased from Sigma-Aldrich (St. Louis, MO). Water and all buffers were of Millipore grade and pretreated with Chelex 100 resin to ensure that the aqueous solution was free of heavy metals. All chemicals were used as received without further purification.

**Characterizations.** Transmission electron microscopy (TEM) images were obtained on a FEI T12 microscope operated at an accelerating voltage of 120 kV. Standard TEM samples were prepared by dropping dilute products onto carbon-coated copper grids. Dynamic light scattering and zeta potential analysis were performed on Nano-Zetasizer (Malvern Instruments Ltd.). Flow cytometry studies were done by using BD FACSCalibur four-color analysis cytometer, which is equipped with 488 and 633 nm lasers (Becton-Dickinson, San Jose, CA) and FlowJo analysis software (Tree Star, Ashland, OR). PET and PET/CT scans at various time points *p.i.* using a microPET/microCT Inveon rodent model scanner (Siemens Medical SolutionsUSA, Inc.). Biodistribution studies were performed by measuring the radioactivity in the tissue in a WIZARD^2^ gamma-counter (Perkin-Elmer). *In vivo* NIRF imaging was performed by using an IVIS spectrum *in vivo* imaging system (*Ex*=745 nm, *Em*=800 nm).

**Synthesis of uniform 65 nm sized HMSN.** Procedures for the synthesis of HMSN with a smaller size of ~65 nm were the same as previously described except 50 nm sized dSiO_2_ was used as hard template. For synthesis of 50 nm sized dSiO_2_, Igepal CO-520 (NP-5; 2 mL) was dispersed in cyclohexane (40 mL) in a three-necked flask and stirred for 5 min. Then, 0.28 mL of ammonia was added into the cyclohexane/NP-5 mixture and stirred for 2 h. Afterward, TEOS (350 uL) was added and the mixture was sealed and kept under magnetic stirring for 48 h before addition of methanol to collect the nanoparticles. The dSiO_2_ was washed and dispersed in 20 mL of deionized water.

**Loading HMSN with hydrophilic or/and hydrophobic drugs**. To load HMSN with hydrophilic drugs, *e.g.* DOX, HMSN with a known mass was re-suspended in 0.5 mg/mL of DOX-PBS solution. The mixture was kept under constant shaking for 24 h at room temperature. Afterward, HMSN(DOX) was collected by centrifugation and washed with PBS for 3 times. The loading capacity was calculated by the following equation: loading capacity % = Amount of DOX in HMSN/Mass of HMSN*100.

To load HMSN with hydrophobic drugs, *e.g.* SUN, HMSN with a known mass was re-suspended in 1 mg/mL of SUN dimethyl sulfoxide solution. The mixture was kept under constant shaking for 24 h at room temperature. Afterward, HMSN(SUN) was collected by centrifugation and washed with water for 3 times. The loading capacity was calculated by the following equation: loading capacity % = Amount of SUN in HMSN/Mass of HMSN*100.

To load HMSN with both hydrophobic and hydrophilic drugs, HMSN with a known mass was first loaded with SUN in dimethyl sulfoxide solution and then continue to be loaded with DOX in PBS. Note that loading DOX first and SUN later will cause the release of DOX in dimethyl sulfoxide solution during the loading of SUN.

**Flow cytometry**. Cells were first harvested and suspended in cold PBS with 2 % bovine serum albumin at a concentration of 5×10^6^ cells/mL, and then incubated with fluorescein conjugated HMSN-ZW800-TRC105 (50 nM) or fluorescein conjugated HMSN-ZW800 (50 nM) for 30 min at room temperature. The cells were washed for three times with cold PBS and centrifuged for 5 min. Afterward, the cells were analyzed using a BD FACSCalibur four-color analysis cytometer, which is equipped with 488 and 633 nm lasers (Becton-Dickinson, San Jose, CA) and FlowJo analysis software (Tree Star, Ashland, OR). “Blocking” experiment was also performed in cells incubated with the same amount of fluorescein conjugated HMSN-PEG-TRC105 (50 nM), where 500 μg/mL of unconjugated TRC105 was added to evaluate the CD105 specificity of fluorescein conjugated HMSN-PEG-TRC105.

**4T1 murine breast cancer model**. All animal studies were conducted under a protocol approved by the University of Wisconsin Institutional Animal Care and Use Committee. To generate the 4T1 tumor model, 4 to 5 week old female BALB/c mice were purchased from Harlan (Indianapolis, IN, USA), and tumors were established by subcutaneously injecting 2 × 10^6^ cells, suspended in 100 μL of 1:1 mixture of RPMI 1640 and Matrigel (BD Biosciences, Franklin Lakes, NJ, USA), into the front flank of mice. The tumor sizes were monitored every other day, and the animals were subjected to *in vivo* experiments when the tumor diameter reached 5-8 mm.
